# Ligation of the Carotid Artery for Injury

**Published:** 1876-03

**Authors:** E. T. Dorland


9 ART. III.—*Ligation of the Carotid Artery for Injury.* Report of Case by E. T. DORLAND, M. D.

The occurrence of a case like the following being, I judge, exceedingly rare, and in some of its features very interesting, I send it for publication. For a still more important reason I deem it worthy of reporting, in that it demonstrates the practicability of ligating some of the larger arteries of the trunk in secondary hemorrhage. In Vol. 1st, page 266, Ericksen, writing in the subject of secondary hemorrhage after the ligation of arteries in their continuity, states, "If the artery be situated in the trunk, as the subclavian, carotid or one of the iliacs, there is nothing to be done but to trust the plugging of the wound, and, in a great majority of cases, the patient will die, exhausted by repeated hemorrhage."

The patient, Mathias Lavyea, was injured, Aug. 27th, 1875, at the Erie Railway Shop. The injury consisted of a fracture of the malar, superior maxillary and sphenoid bones. The patient was first visited by Dr. Mixer, who applied a compress and bandage to hold the malar bone up in place. The patient improved rapidly, and was able to be out in a few days from the receipt of injury. Dr. Lynde took charge of the case on the 30th of August. The pupil of the left eye was dilated and the pulse was irregular. These conditions continued from the day on which he was injured until his death. On September 10, a pulsating tumor made its appearance under the left eye, completely filling the antrum and lifting the malar bone. This tumor rapidly increased in size, and a consultation was held, consisting of Drs. Mixer, Briggs, Harding, Oldfield and the writer. Preparation had been made for the ligation of the carotid, but it was thought best to postpone the operation a few days and watch developments. On the 13th, 14th and 16th of September, arterial hemorrhage occurred from the nostrils. On the night of the 16th this hemorrhage came near being fatal.

On the morning of the 17th of September, Drs. Lynde and Briggs plugged the nostrils anteriorly and posteriorly. This failed to control the hemorrhage, and on the afternoon of September 18, Dr. Lynde, assisted by Drs. Mixer, Baker, Briggs, Harding and myself ligated the primitive carotid, after finding that pressure on the internal and external carotid separately failed to control the pulsation. The patient was comfortable for the next two weeks, excepting that he suffered with a severe diarrhœa, which came on the third day after the operation, and lasted for three or four days. On the tenth day after the operation, the wound was entirely healed, except at the point of exit of the ligature.

At this time, Dr. Boardman kindly visited the case, and observing the condition of the patient, suggested that a strong light be brought before the eye to test the susceptibility of the retina to impressions. The pupil was but little changed from its previous condition by the test. Similar experiments, subsequently made, were invariably followed by the same results.

The objects for which the ligation was undertaken were fully accomplished, viz: the arrest of the development of the aneurismal tumor, and the control of the nasal cavity.

Excepting a slightly irregular pulse and the dilated pupil (as before mentioned), the patient appeared now entirely well. Not a symptom had occurred during this time to indicate that the brain had suffered from the ligation of the artery. On the morning of the fourteenth day after the operation slight arterial hemorrhage took place from the wound at the site of the ligation. This hemorrhage increased in the afternoon, and at three o'clock Drs. Lynde, Harding and Wasson etherized the patient and made preparation to open the wound and reapply the ligature if necessary. But traction on the ligature failing to remove it or to produce auy increase of the hemorrhage, the patient was placed in bed and hopes entertained that the bleeding came from some small vessel. No more hemorrhage took place from the wound until 7 P. M., when it was decided by Drs. Lynde, Harding and myself to apply a compress. At midnight the hemorrhage became more profuse, and when partially controlled by pressure with the fingers, the areolar tissue became infiltrated with blood. So rapidly did this infiltration take place, that before preparation could be made to open the cicatrix and apply the ligature, the tumor had attained a size nearly equal to a man's fist. Dr. Lynde at once laid open the wound. The bleeding was found to come from the ligated vessel. The ligature, being slightly pulled on, came off, followed by a terrific hemorrhage from both ends of the artery. In the distended condition of the soft parts, this hemorrhage could not be materially lessened by pressure above and below the point of ligation, but by placing the finger directly upon the artery, the hemorrhage was controlled. The finger being kept in place upon both ends of the artery, a large tenaculum was made to penetrate the walls of both these ends, and by as strong traction on the tenaculum as the coats of the vessel would admit without rupture, the hemorrhage was so far controlled and the ends of the vessel so far elevated as to admit the passage of the aneurism needle; first under the proximal end, and ligation of this being accomplished, the distal end was secured in like manner. The wound was now closed by the interrupted suture, and the patient allowed to rest, after suffering the most frightful hemorrhage I ever witnessed at a surgical operation. The next morning we found our patient comfortable, and without an untoward symptom. He continued to do well for eleven days, when a profuse hemorrhage again occurred in the night, but this time only from the distal end of the artery. Dr. Lynde, assisted by Dr. Harding and myself, by lamplight, etherized the patient, opened the wound (which was healed), and again proceeded to ligate. The artery, giving way up to the point of bifurcation into external and internal carotid, required two ligatures—one for each branch. These having been applied by means of the tenaculum and an aneurism needle, the wound was closed with interrupted sutures, and the patient put into a comfortable position and allowed to rest for the remainder of the night. The next morning we found our patient comfortable. Without one unpleasant symptom (always excepting pulse and pupil), he passed the next two weeks happy and hopeful. At the expiration of this time, the ligatures came off without a stain of blood. The patient was now apparently well, sitting up most of the time, with good appetite, and inquiring when he could go to work. He continued to improve until the 3d of November, when, without one premonitory symptom, he had a severe chill, followed by high fever, pain in the head, delirium, coma and death.

A post mortem examination was made on the 6th of November by Drs. Lynde, Mixer, Kamerling, Dagenais, Harding and myself. The following notes of the post mortem were furnished by Dr. Harding: Dura mater congested and adherent to pia mater. The latter was clouded with lymyh and pus. The ventricles of the brain contained serum and pus—conclusive proof of inflammation. The brain itself was healthy in appearance. There was a fracture of the left greater ala of the sphenoid bone, extending into the left superior maxillary. The malar bone was also badly fratured. The left primitive carotid was filled below the point of ligation with a plug of lymph and blood. The wound made by the operation was healed. The other vessels, and the nerves in proximity to the artery, were uninjured and healthy.

